# Geographically Modified PageRank Algorithms: Identifying the Spatial Concentration of Human Movement in a Geospatial Network

**DOI:** 10.1371/journal.pone.0139509

**Published:** 2015-10-05

**Authors:** Wei-Chien-Benny Chin, Tzai-Hung Wen

**Affiliations:** Department of Geography, National Taiwan University, Taipei, Taiwan; Universidad Rey Juan Carlos, SPAIN

## Abstract

A network approach, which simplifies geographic settings as a form of nodes and links, emphasizes the connectivity and relationships of spatial features. Topological networks of spatial features are used to explore geographical connectivity and structures. The PageRank algorithm, a network metric, is often used to help identify important locations where people or automobiles concentrate in the geographical literature. However, geographic considerations, including proximity and location attractiveness, are ignored in most network metrics. The objective of the present study is to propose two geographically modified PageRank algorithms—Distance-Decay PageRank (DDPR) and Geographical PageRank (GPR)—that incorporate geographic considerations into PageRank algorithms to identify the spatial concentration of human movement in a geospatial network. Our findings indicate that in both intercity and within-city settings the proposed algorithms more effectively capture the spatial locations where people reside than traditional commonly-used network metrics. In comparing location attractiveness and distance decay, we conclude that the concentration of human movement is largely determined by the distance decay. This implies that geographic proximity remains a key factor in human mobility.

## Introduction

### Background

The real world contains extensive connections. Ecologists believe that there is one ecosphere for all living organisms, that what affects one affects all, and that everything is connected to everything else [1]. Geographers extend this fundamental concept to connected spatial features located in finite geo-spaces [2]. The elements of a terrestrial system are connected, and their spatial relationships should not be ignored. A network approach, which simplifies geographic settings into combinations of nodes and links, emphasizes the connectivity and relationships among spatial features [3]. The nodes represent spatial features that can be indicated as points (e.g., ports, airports, or buildings) or areas (e.g., countries, cities, or regions). The links are formed by connections or volumes of flow between locations, for example, commuting volumes between regions or volumes of air traffic between airports. Analysis of the structure of a flow network can be used to elucidate interactions among these features. To explore and understand spatial features and the structures within them, recent studies have adapted network topological analysis frameworks to geography. Alderson and Beckfield [4] created a world-city network in which the nodes were world cities and the links were formed by interactions among multinational enterprises and their subsidiaries in different cities; they explored the economic status and position of each city within the network. El-Geneidy and Levinson [5] and Reggiani et al. [6] created a commuting network to determine accessibility. Jiang [7], Jiang et al. [8], and Jiang and Jia [9] created a street-to-street topological network to elucidate human movement on streets. Wang et al. [10] created a street network and used this network to explain the relative importance of different locations with respect to land-use. Ducruet et al. [11] created an inter-port network to measure the vulnerability of each port. Guimera et al. [12], Reggiani et al. [13], Ducruet et al. [14], and Scholz [15] used airline networks to explore the concentration of air transportation and find hubs or hot spots. In summary, these studies created topological networks of spatial features and used them to explore geographical connectivity and structures. These studies have focused on the network positions of connected spatial features and on the vulnerabilities and strengths of locations within networks.

Previous studies have used network metrics to retrieve information from the real world. The concept of network centrality has often been used to explore locational characteristics in connection with interactions within the network [12] [13] [14] [15]. Alderson and Beckfield [4] examined interactions among multinational enterprises and their subsidiaries across cities, using out-degree centrality to show the influence of each city on the world economy, in-degree centrality to show each city’s ability to attract investment from other cities, closeness centrality to show each city’s independence, and betweenness centrality to show the potential of each city to act as a bridge, brokering interactions (investment flow) among cities or subgroups of cities. However, network centrality does not capture the transitive effect of network topology and thus could underestimate or overestimate the importance of nodes. PageRank [16], an algorithm used by the Google Search Engine, is another useful network metric used to identify important web-pages. The PageRank (PR) algorithm uses an iterative calculating process involving simulation of the movement of random surfers within a web-page network linked by hyperlinks. The algorithm is used to identify highly recommended locations where people may tend to gather. In other words, PageRank could quantify the accessibility of locations potentially reached by people. As a result, the PageRank algorithm has been used to help identify important places visited by people or automobiles in the geographic literature [5] [7].

Geographic proximity and location characteristics are important factors in measuring the importance of locations [3] [17] [18]. However, the PageRank algorithm focuses on network topological relationships, such as connectivity and transitivity, while neglecting geospatial structures in networks, including geographical proximity. Geographic studies emphasize the intensity of spatial interactions, where intensity is viewed, in most geographic models, as a function of geographic distance and the attractiveness of locations [17]. These concepts are also used to measure geographic accessibility and population mobility [19] [20] [21]. However, most of these geographic models ignore the effects of spatial network topology [3]. Therefore, the objective of this study is to propose geographically modified PageRank algorithms, which are based on distance-decay characteristics and gravity function, to integrate geographic factors into network metrics used to identify important locations in geospatial networks. We also attempt to examine the robustness of the algorithms in both national-scale intercity network and city-scale connections, with different model functions and parameter settings.

### PageRank algorithm: Basic concepts and its extensions

**Fig 1:**
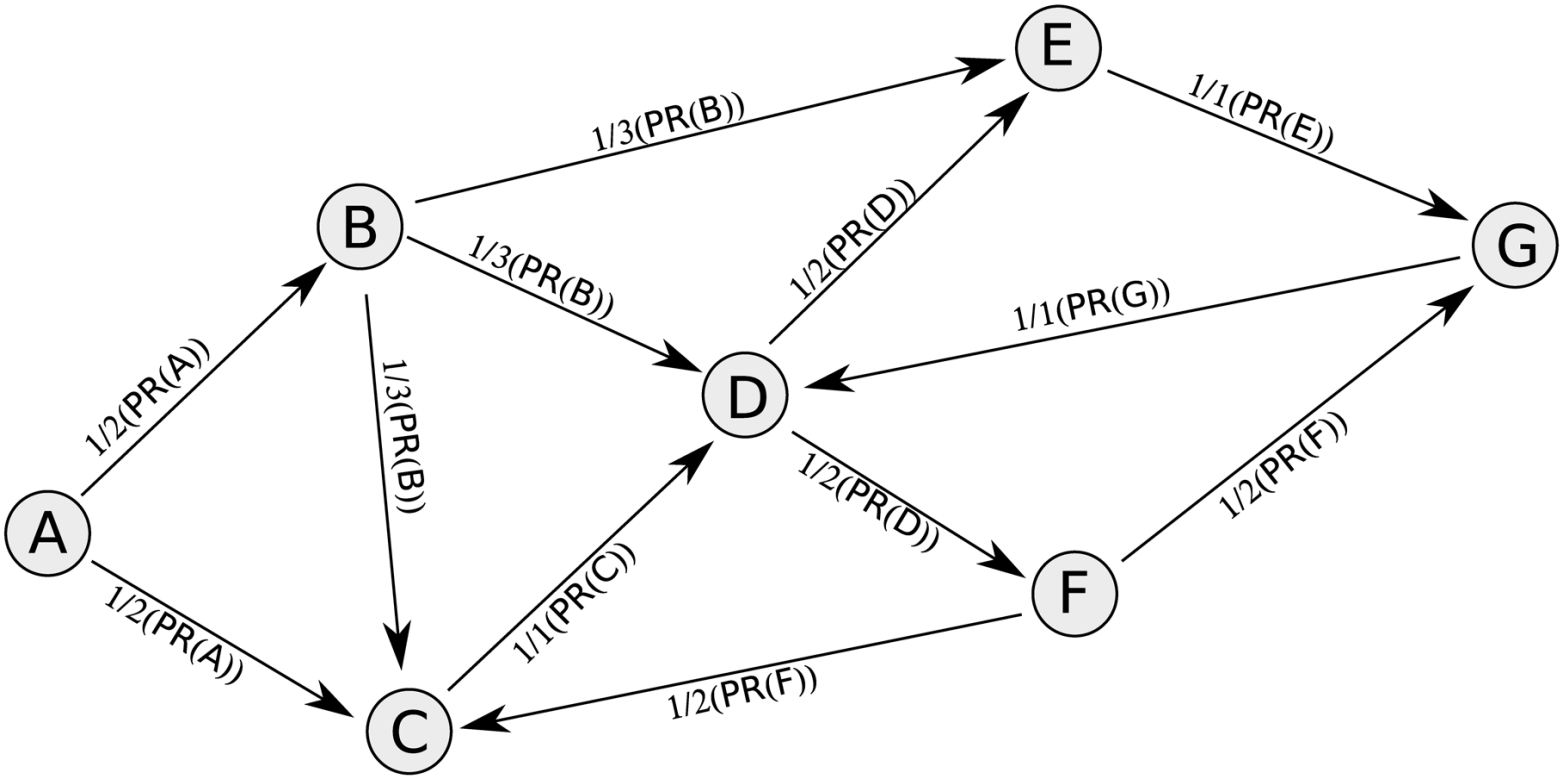
An illustration of PageRank algorithm. The probabilities of a random surfer browsing from each web-page to the outgoing web-pages are assumed to be equal.

The PageRank algorithm can be regarded as a procedure for simulating the movement of random surfers within a web-page network connected by hyperlinks [16]. Analysis of the movement of random surfers based on the probability of transfer from one web-page to another (Fig 1) indicates the distribution of random surfers among web-pages.

PageRank analyzes the movement of random surfers by measuring the probabilities of movement between nodes. At the beginning, all nodes are assigned an equal PR score. The score is then transferred between nodes based on iterations within the links structure of the network. After a sufficient number of iterations in the movement, the PR score attains an equilibrium value, and the distribution of the PageRank score indicates the distribution of random surfers as a static result. One of the main procedures is to measure the proportion of PR scores that flow on each link, as determined by the link structure. The proportion of PageRank scores that move on each link is calculated by dividing the PageRank score of the source node by the number of out-links of the source node (Fig 1). That is, if a node has three out-links (node B), the PR score for movement on each out-link of the node would be 0.33 of its PR score. The PR score for movement on each link would also become equilibrium while the nodes attained equilibrium PR scores.

Besides moving along the network connection, a surfer might also stop browsing a page and start visiting a random page, which is a moving behaviour outside of the hyperlink connection. However, in a geospatial network, people move physically between spaces. Thus, the movement outside of the network can be assumed to not take place in a geospatial network study [5]. In other words, in the calculation of PR algorithm, we could assume that all of the people move along the network connections.

Attractiveness, an internal factor of nodes, is a way of quantifying the power of each target node to draw random surfers from its incoming links. To weight the nodes with attractiveness, Xing and Ghorbani [22] proposed a Weighted PageRank (WPR) algorithm, which uses the in-degree of each node to determine its attractiveness and compared the attractiveness of the target nodes for each source node. The PR score sent to each target nodes is then proportional to its relative attractiveness. Therefore, a node with a low attractiveness value could be comparatively less attractive than a comparing node with a higher attractiveness value.

Some studies used population flow properties to extend PR algorithms for identifying the attractiveness of a location, such as flows of workers [5] or flows of migrations among areas [23]. El-Geneidy and Levinson developed the PlaceRank algorithm [5] incorporating the numbers of workers to measure the cumulative opportunity of each location in terms of job seeking. Zhong and Liu [23] used proportions of migrations and distances between origin and destination cities to measure the attraction score of each city for long-term living. To measure the potential flows and the spatial concentration of human movement, origin-destination flow data (e.g. ridership or inter-townships mobility statistics), is required for these extended PR algorithms. These data could be collected and obtained in some well-developed cities, such as RATP’s Paris transport system [24] or oyster data from the London transport system [25] [26]. However, in developing countries, flow data is often difficult to collect comprehensively. The intention of our proposed algorithms is to capture the spatial concentration of population movement by only considering the topology of the network and geographic factors rather than origin-destination network flow.

## Methods

### Distance-Decay PageRank (DDPR): Incorporating the effect of geographic proximity

One major geographic consideration in approaching a location is the travel cost. Because each terrestrial location has a fixed coordinate, approaching a destination from a point of origin would require a corresponding travel cost associated with the journey. The intensity of spatial interactions between locations decreases as travel cost increases. This is the distance-decay effect [3] [18]. The geographic distance between nodes can be regarded as one of the forms of travel cost in a geospatial network. It could be an internal factor of the links or an external factor of the nodes and thus be determined by the locations of both nodes.

We incorporated geographic distance between nodes as a travel cost in the PageRank algorithm, specifically, in the Distance-Decay PageRank (DDPR) algorithm. To capture the distance-decay effect, we used the inverse distance between target nodes and the source node and sent the PR score from the source node to its target nodes proportional to the inverse distance (see Eqs (1) and (2)). For each node *a*, which has several incoming links that come from a set of nodes *b* (*b* ∈ *I*_*a*_); for each node *b*, which has several outgoing links that target a set of nodes *c* (*c* ∈ *O*_*b*_, and *a* ∈ *O*_*b*_).

$$DDPR_{t}\left(a\right)=\sum_{b\in I_{a}}DDPR_{\left(t-1\right)}\left(b\right)\times \frac{F_{DD}\left(b,a\right)}{\sum_{c\in O_{b}}F_{DD}\left(b,c\right)}$$ (1)

$$F_{DD}\left(i,j\right)=\frac{1}{distance{\left(i,j\right)}^{\beta }}$$ (2)

where *DDPR*_*t*_(*a*) is the PR score for node *a* at the iteration *t*, *β* is the distance factor, and *distance*(*i*, *j*) is the distance between the pair of node *i* and node *j*.

The distance factor, *β* in Eq (2), determines the scale of the distance-decay effect. The larger the distance factor, the steeper the distance-decay curve and the more significant the distance-decay effect will be. A distance factor of 0 implies that the distance-decay effect does not exist, and the result would be similar to the one obtained from the original PageRank algorithm. The distance-decay relationship was formulated as a power-law decay (inverse-distance) function in Eq (2). Sensitivity analysis for measuring the effect of distance factor in Eq (2) on the model performance was further assessed. Moreover, some studies suggested to use exponential functions for modeling distance-decay relationship [27] [28] [29] [30]. We also compared the power-law distance-decay function with the exponential-decay functions on model performance.

### Geographic PageRank (GPR): Incorporating effects of geographic proximity and attractiveness of locations

Geographic considerations often consist of attractiveness and impedance among locations. The intensity of the spatial interactions between locations is assumed to be proportional to their attractiveness to one another and inversely proportional to their impedance [17] [18]. In geographical science, attractiveness refers to the force that pulls people to a target destination whereas impedance refers to the movement resistance that are required to be overcome [31]. In a geospatial network, attractiveness and impedance affect people’s movements among the nodes, and these effects could be captured by calculating the probability of movement through each link. We propose a modified PageRank algorithm, the Geographical PageRank (GPR) algorithm, which includes the concepts of attractiveness and impedance. Similar to the concept of gravity modeling, we assume that the probability people choose nearer and more attractive locations to be higher. In other words, people may wander around linked nodes that are close to one another, but they may also be willing to travel to more distant but more attractive nodes. Xing and Ghorbani [22] suggested that the incoming links could represent the direct popularity of each node, which were used as the weights of comparison in WPR [7]. Therefore, in GPR, we establish a function of spatial interactions that consists of the in-degree of each target node (*a*), which represents attractiveness, and distance from its source node *b*, which represents impedance (see Eq (3)). We then use this score, *F*_*G*_, as a basis for comparing the target node with its comparing nodes (*O*_*b*_) (see Eq (4)). Thus, a higher PR score would be sent to a nearer and/or a very attractive node. Because GPR combines the attractiveness function and the distance-decay function, the target node’s attractiveness and the distance between the target node and the source node are considered simultaneously, implying that spatial interactions between nodes decay with distance and increase with attractiveness. The mathematical explanation of GPR is as follows.

$$F_{G}\left(i,j\right)=\frac{indegree{\left(j\right)}^{\alpha }}{distance{\left(i,j\right)}^{\beta }}$$ (3)

$$GPR_{t}\left(a\right)=\sum_{b\in I_{a}}GPR_{\left(t-1\right)}\left(b\right)\times \frac{F_{G}\left(b,a\right)}{\sum_{c\in O_{b}}F_{G}\left(b,c\right)}$$ (4)

where *GPR*_*t*_(*a*) is the GPR score of the target node *a* at the iteration *t*, *indegree*(*j*) is the in-degree of the target node *j*, *α* is the exponent of the in-degree. *distance*(*i*, *j*) is the distance between pair of node *i* and node *j*, and *β* is the distance factor. Similar to the DDPR, a sensitivity test on the distance factor and a comparison between the distance-decay curve was studied in the following section.

**Fig 2:**
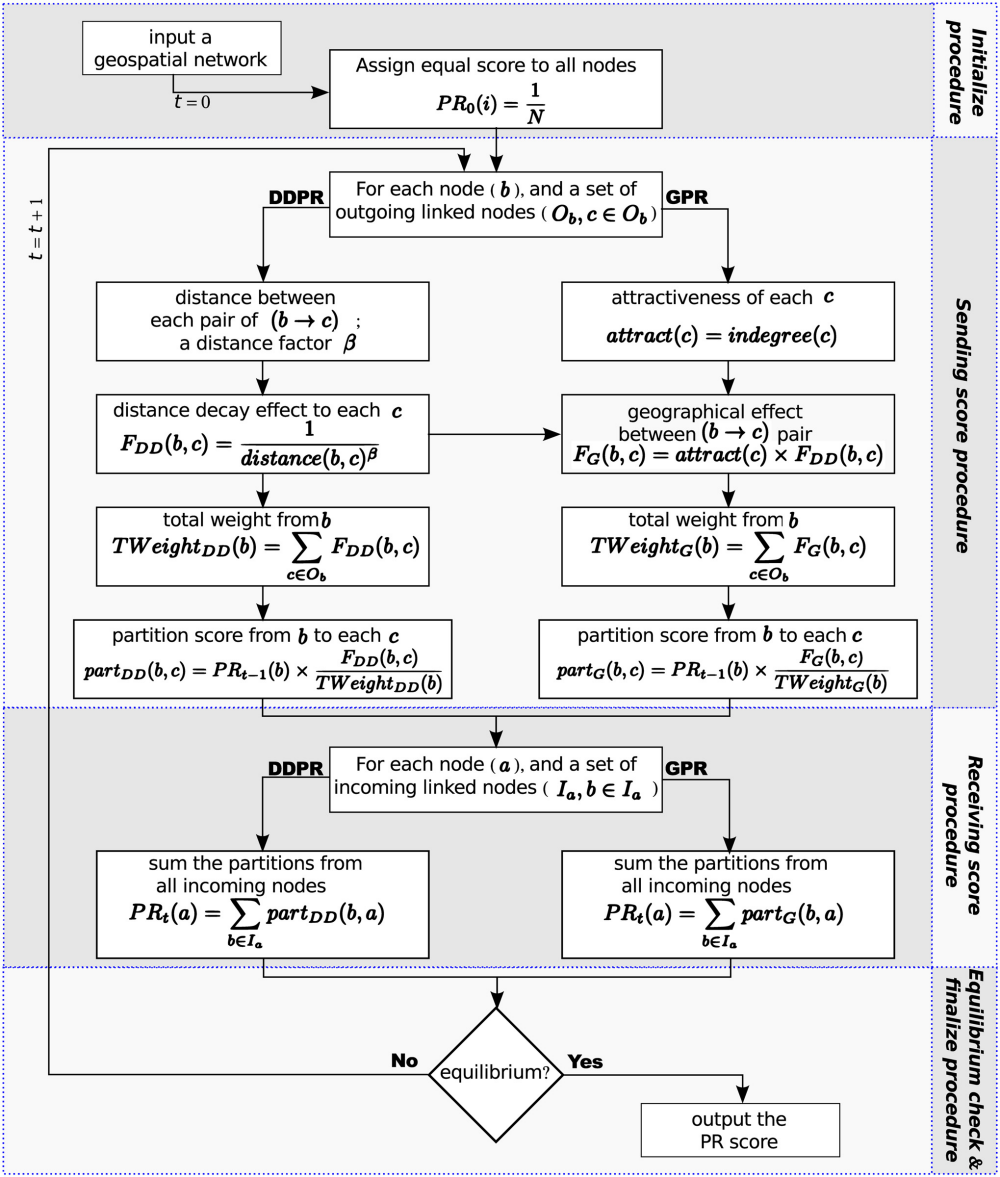
The framework of DDPR and GPR algorithms.

In summary, the proposed algorithms of both DDPR and GPR consist of four components (see Fig 2). These include (1) Initialization: at the beginning, all nodes in the geospatial network are assigned an initial score; (2) Sending score procedure: for each source node (*b*), the distance to each of its target nodes *c* and the attractiveness of each target node are used to calculate the function of the distance-decay effect (for DDPR) and the spatial interaction effect (for GPR); then its score is divided and sent to each of its target nodes, according to the proportion calculated using the two functions; (3) Receiving score procedure: when all nodes have sent out their previous iteration score, each node *a* also receives scores from its linked nodes *b*, which are summed and used as its current score; (4) Equilibrium checking: if all of the nodes’ current scores are same as their previous scores, the system has reached a state of equilibrium, and the current score is then the output, i.e., the final PR score; if not, the system would return to the sending score procedure and start the next iteration.

### Sensitivity of choosing distance-decay functions

Distance-decay relationship is often formulated as power-law or exponential functions in geographical literatures [3]. With an exponential-decaying function, the strength of interaction between nodes would decrease more dramatically with increasing distance [27] [28] [29] [30]. There are two major components that influence distance-decay relationship: the shape of distance-decay curves (power-law vs. exponential functions), and the parameters of the distance-decay functions. The different settings of these components may affect the distribution of DDPR and GPR scores, therefore, we established three experiments to assess the sensitivity of choosing distance-decay functions and their parameters.

First, we performed an experiment of setting different values of the *β* between a range from 0.0 to 3.0 with 0.1 increment in Eqs (2) and (3). These results were compared by their correlation coefficient (*rho*) with the index of concentration of human movement to assess the robustness of the model results. Second, we performed another experiment on the exponential form of distance-decay functions in DDPR and GPR (Eqs (5) and (6)). We defined a distance-control factor (*d*_*γ*_) to assess the shape of exponential-decay function. The range of *d*_*γ*_ is between the shortest (*d*_*min*_) and longest (*d*_*max*_) distance between two connected nodes. We created a subscripted variable *γ* with range from 0 to 1 Eq (7). The parameter settings of *γ* were used for sensitivity analysis to investigate the impact of the distance-control factor on the correlation between spatial concentration of human movement and the modified PR scores (DDPR and GPR) with exponential decay function. The values of *γ* were in range from 0.00 to 1.00 with 0.05 increment. The higher *d*_*γ*_ would lead to a higher value of $F_{DD}^{\prime }$ or $F_{G}^{\prime }$, and it means the distance-decay effect is less significant. Third, the two distance-decay functions in DDPR and GPR were compared. Eqs (2) and (3) are the power-law distance-decay functions for DDPR and GPR, respectively. Eqs (5) and (6) are the exponential distance-decay functions for DDPR and GPR, respectively.

$$F_{DD}^{\prime }\left(i,j\right)=\frac{1}{e^{distance\left(i,j\right)/d_{\gamma }}}$$ (5)

$$F_{G}^{\prime }\left(i,j\right)=\frac{indegree{\left(j\right)}^{\alpha }}{e^{distance\left(i,j\right)/d_{\gamma }}}$$ (6)

$$\gamma =\frac{d_{\gamma }-d_{min}}{d_{max}-d_{min}}$$ (7)

## Results

### Case Study 1: national-scale intercity network

**Fig 3:**
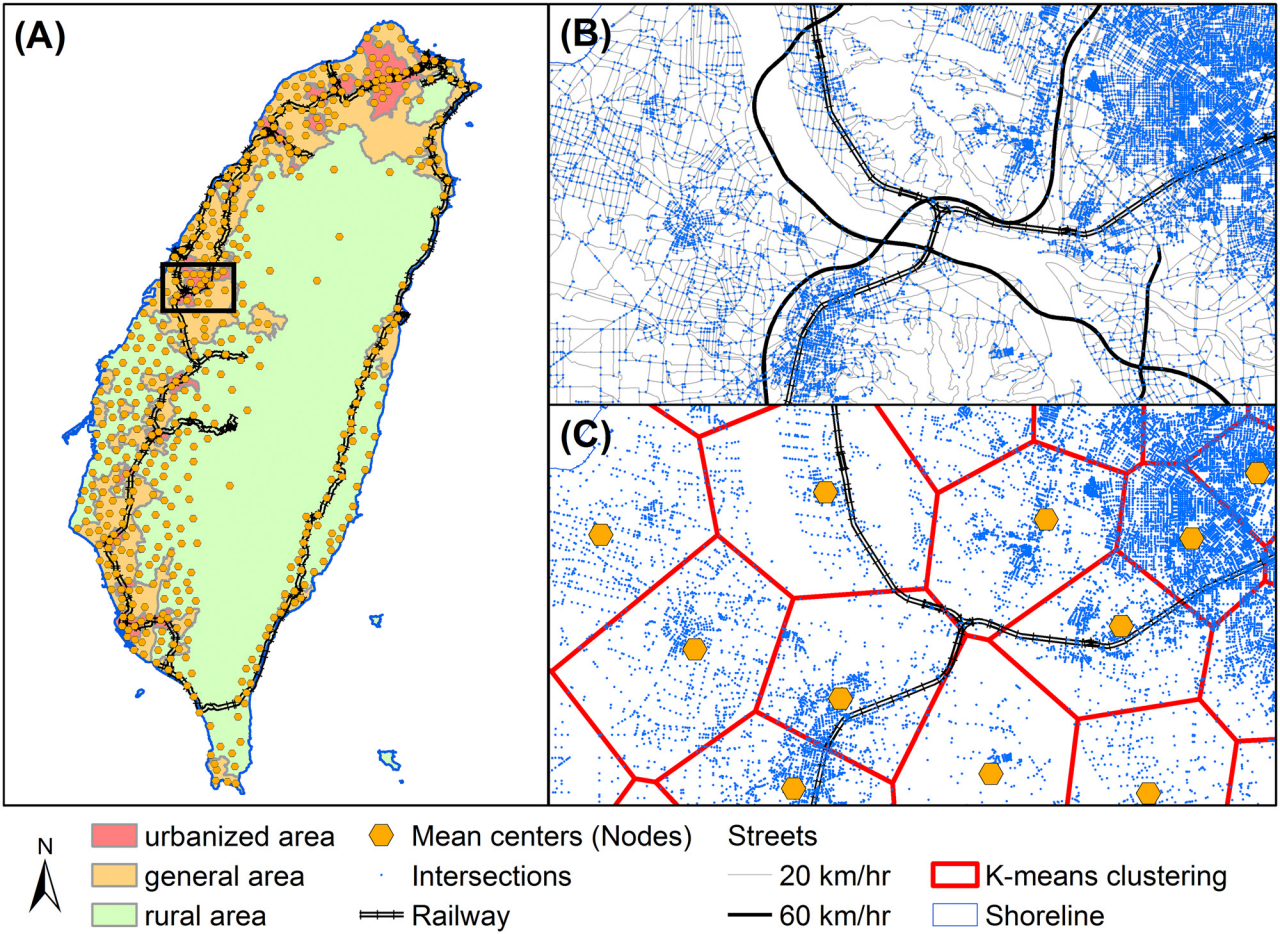
Transformation from transportation system to geospatial network. Spatial distribution of (A) national-scale population centers and urbanization status, (B) junctions of the streets, and (C) the centroid nodes where people gathering together. Data souce: Institute of Transportation, MOTC (Taiwan).

The national-scale case study area was the Taiwan Island, which has a population of approximately 22.6 million. We transformed the Taiwan Island transportation layers into a geospatial link-node network of national-scale intercity relationships. The network consists of nodes, which are defined as population centers where people reside, and links between nodes are defined as connections between settlements. Links represent the possibility of moving between settlements in one hour of travel time. The national-scale transportation system includes all levels of street and railway networks [32]. We used k-means clustering procedure to group the streets’ junctions (a total of 391,588 junctions) to identify the centroid nodes where people agglomerate. The k-value (number of node) is selected based on the total population of Taiwan. In this study, we chose one node to represent around 50 thousand people. Because Taiwan’s total population is around 22.6 million, the k-value was set to 500 (Fig 3). The travel time between each node through the street and railway networks was then calculated, and a link was established if two nodes were reachable from both directions within one hour [33]. The minimum and maximum distance between two nodes are 2.95 km and 83.26 km, respectively. The intercity network was then used for the calculations of the PR algorthms.

To evaluate the performance of the proposed network metrics in capturing important nodes in a geospatial network, we used township-level indices, including population density, the densities of total and incoming daily automobile flow for each township, as surrogates for the concentration of population movement. We also compared the performance between three urbanization status [34]. The urbanization status was divided into three classes, including urbanized area, general area (newly developed and ordinary area), rural area (aging, agro-township and remote area). Population data was obtained from the Monthly Statistics of the Ministry of the Interior, Taiwan [35]. Daily automobile flow data was obtained from the Institute of Transportation of the Ministry of Transportation and Communication [36], which has studied inter-township human flow for the year 2005. Township-level population density indicates where people reside, capturing the spatial concentration of population. Inter-township total automobile flow (total incoming and outgoing) shows the moving intensity (or busyness) of each township, and the proportion of incoming automobile flow shows which townships are most attractive to people. The correlations between the three township-level indices were shown in Table 1.

**Table 1:** The correlation between the population density and the densities of total and incoming daily automobile flow for each township.

	*Population*	*Total* − *flow*	*In* − *flow*
*Population*	1.000		
*Total* − *flow*	0.881***	1.000	
*In* − *flow*	0.878***	0.999***	1.000

*** significant at 0.001 level.

**Fig 4:**
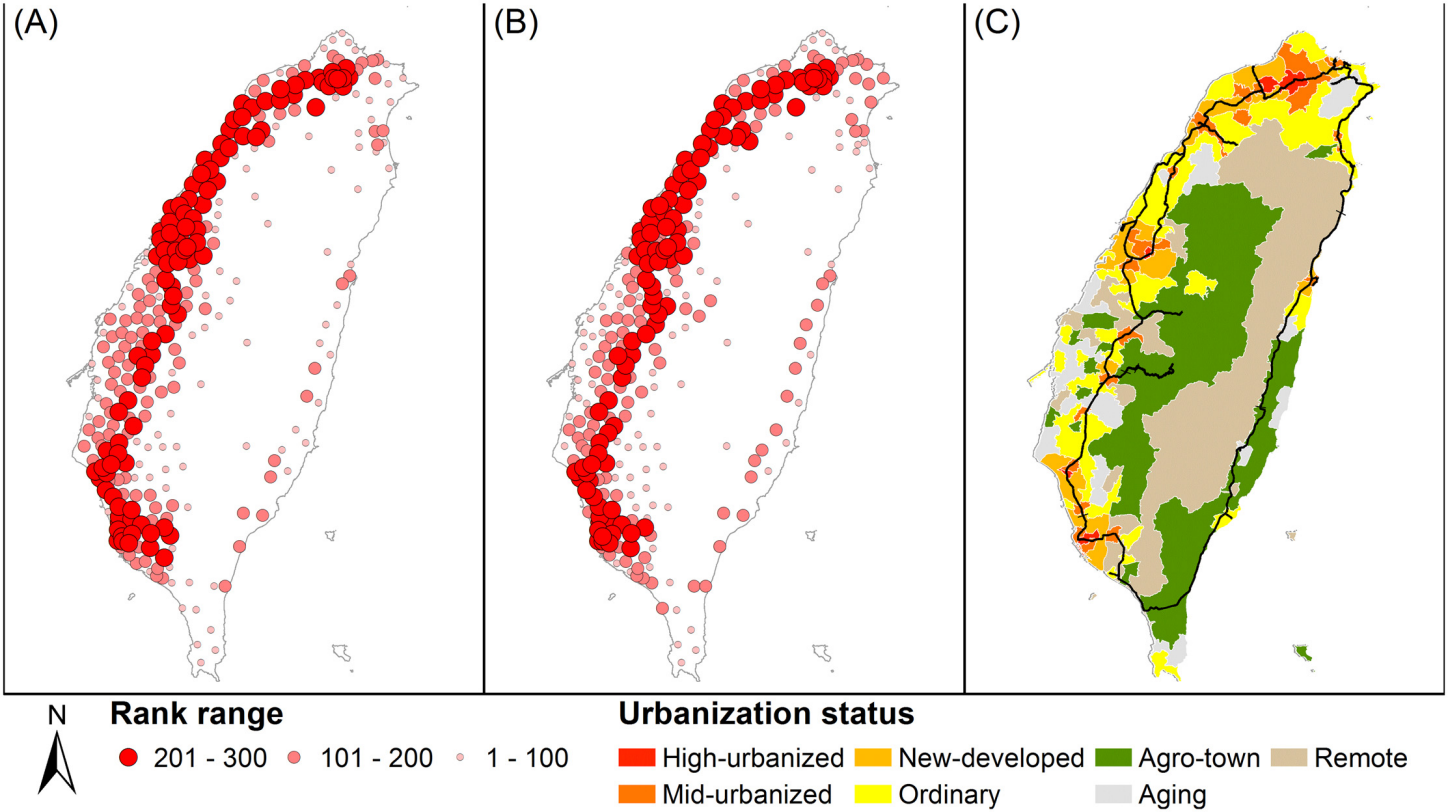
Spatial distributions of the: (A) DDPR and (B) GPR, and (C) urbanization status.

The parameters, *α* and *β*, in GPR/DDPR algorithms were both set to 1 for initially assessing their performance in the national scale. The spatial distributions of the DDPR and GPR with urbanization status are shown in Fig 4. The nodes with larger symbols are higher-ranking nodes, implying that such nodes may be characterized by a higher concentration of human movement than lower-ranking nodes. The maps of the DDPR and GPR show similar spatial patterns. Higher-ranking nodes are located in more heavily urbanized areas. Spatial patterns of DDPR and GPR also indicate hierarchal structures in which lower-ranking nodes are surrounded by higher-ranking nodes. The lowest-ranking nodes are located around agricultural, aging, and remote town areas.

We then compared the correlations between the rankings of nodes for DDPR, GPR, degree centrality and the concentration of human movement, as shown in Table 2. The results indicate that among all the network metrics, DDPR has the highest correlation with the indices of concentration of human movement (*rho* = 0.67–0.71), followed by GPR, which has the second highest correlation (*rho* = 0.60–0.63). The correlation results of PR are similar to the degree. This is because the geospatial network is established as an undirected network [37].

**Table 2:** The spearman rank’s correlation between the PR algorithms, degree centrality and the concentration of human movement.

	*Degree*	*PR*	*WPR*	*DDPR*	*GPR*
*Population*	0.566***	0.567***	0.573***	0.713***	0.627***
*Total* − *flow*	0.508***	0.510***	0.530***	0.671***	0.605***
*In* − *flow*	0.507***	0.510***	0.529***	0.669***	0.604***

*** significant at 0.001 level.

**Fig 5:**
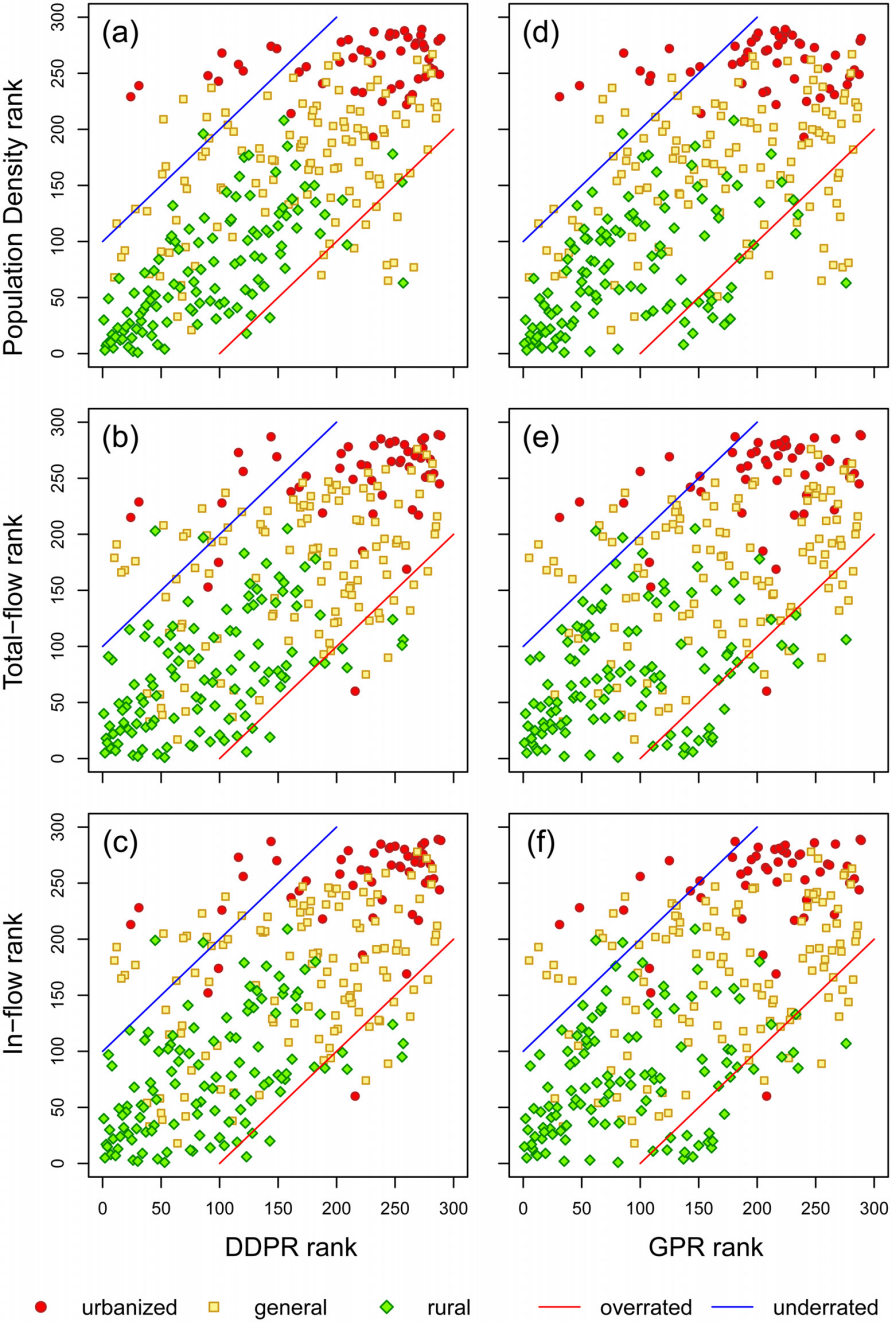
Association between DDPR (a-c) and GPR (d-f) ranks with human movement concentration ranks. (a) DDPR: population density; (b) DDPR: in-flow density; (c) DDPR: total flow density; (d) GPR: population density; (e) GPR: in-flow density; (f) GPR: total flow density. Most urban areas are clustered in the upper-right area of each plot in the figure, indicating that they are rated as having high DDPR and GPR rankings and as areas with high-concentration of human movement. On the other hand, most rural area nodes are clustered in the bottom-left areas of the plots, indicating that such areas are rated as having low DDPR and GPR rankings and as areas with low-concentration of human movement. Most nodes in the upper-left area of the plot are urbanized areas, which suggests that the ranks of high-level urbanized areas could be underrated by the DDPR and GPR scores.

Fig 5 shows the ranks of DDPR and GPR and the corresponding ranks of concentration of human movement. Comparing the differences between the ranks of DDPR with the three indices of human movement concentration, we calculated the average rank difference between DDPR and each of the three indices, finding range between 47 to 53. When comparing the GPR with the three human movement concentration indices, the average rank differences ranges between 56 and 60. In Fig 5(a)–5(c), more than half of the nodes have DDPR ranks with patterns similar to those of the human movement concentration indices. In Fig 5(d)–5(f), more than half of the nodes are shown to have GPR rankings similar in pattern with human movement concentration indices.

We defined the mis-rated townships as those whose rank-differences is higher than +100 (overrated) or lower than -100 (underrated) (Fig 5). The ranks-differences of a township were calculated by subtracting its human movement indices ranks by its DDPR/GPR ranks. Points that fall on the upper-left side of the blue line in Fig 5 represented the underrated townships. Points that fall on the bottom-right side of the red line represented the overrated townships. Among the three classes of urbanization status, rural areas have the lowest mis-rated perentage (5.1% to 9,3% for DDPR and 15.3% to 18.6% for GPR); urbanized areas have the second lowest (15.7% to 17.6% for both DDPR and GPR); general areas have the highest mis-rated percentage (17.5% to 20.0% for DDPR and 19.2% for GPR). Since general areas are the transition region between urbanized areas and rural areas, their high mis-rated percentage suggested that transition areas might have other factors that are not captured by topological and spatial structure, such as local resources and developments. However, most of the mis-rated urbanized areas fall outside of the blue line, meaning they were mostly underrated rather than overrated. In contrast, PR and WPR have higher mis-rated percentage (15.7% to 19.6% for urbanized area, 22.5% to 33.3 for general area, and 20.3% to 21.2% for rural area) than DDPR and GPR. The mis-rated percentage in urbanized areas for PR and WPR are similar to the DDPR and GPR. This demonstrate that the topological structure acted as the dominant factor in urbanized areas, and lead to similar failure at capturing the concentration of human movement. However, the mis-rated percentage of PR and WPR are higher than DDPR and GPR in general areas and rural areas, suggesting that, with consideration to their spatial structure, the DDPR and GPR would better associate with the concentration of human movement indices in these areas.

### Case Study 2: city-scale urban network

Three major Taiwanese cities, with different geospatial characteristics, were used to analyze the usefulness of the city-scale urban network in identifying important locations with high concentrations of human movement. Taipei City is the capital and the political and economic center of Taiwan; it has a population of about 2.6 million. Taichung City is a polycentric city that consists of coastal and inner core areas; it has a population of about 2.6 million. Kaohsiung City has the largest harbor in Taiwan; it has a population of about 2.8 million, and its major economic activities are concentrated near coastal areas. By using one centroid node to represent around 50 thousands people, we located 60 nodes from the junctions found in each city (there are 9,244 junctions in Taipei City, 18,728 junctions in Taichung City, and 16,877 junctions in Kaohsiung City). In each city-scale network, the time-threshold for a link relationship within a city was set to 30 minutes, which represents one-way commuting time. The village-level population data in each city used to represent the human movement concentration in city scale were villages level population data was obtained and organized from the districts’ Household Registration Offices website [38].

**Fig 6:**
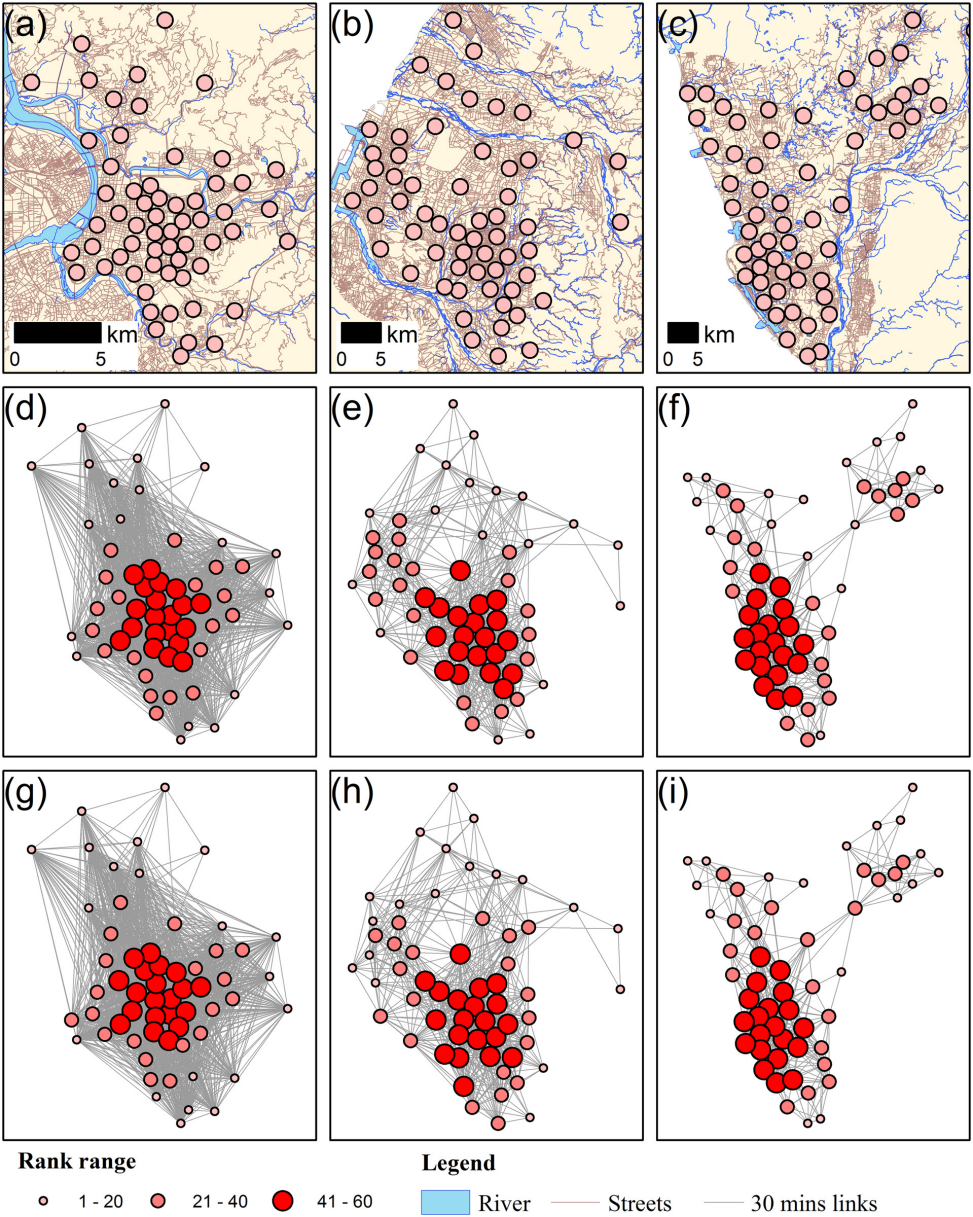
The spatial distribution of nodes with underlying network; the DDPR and GPR ranks results. The spatial distribution of nodes with underlying network for: (a) Taipei city; (b) Taichung city; (c) Kaohsiung city. DDPR ranks result with 0.5 hour transportation network for: (d) Taipei city; (e) Taichung city; (f) Kaohsiung city; and GPR ranks result for: (g) Taipei city; (h) Taichung city; (i) Kaohsiung city. The size of the nodal circle is proportional to the ranks of DDPR or GPR scores. The nodes with higher rankings are concentrated in the central area in Taipei City (d,g), in the southern inner area in Taichung City (e,h), and in the southern coastal area in Kaohsiung City (f,i). These higher-ranking nodes capture the locations of central business district (CBD) among the three cities. The lower ranking nodes are located in the outer rings of Taipei City. The northern Taichung, with lower rankings, is separated from the CBD by a river. In Kaohsiung City, the areas with lower rankings are concentrated in the northern underdeveloped regions. Data souce: Institute of Transportation, MOTC (Taiwan).

To compare the network complexity of the three major city networks, network density was used to differentiate the degrees of connectivity of the different cities. Network density is defined as the ratio of existing links to the maximum possible number of links in a city. Table 3 shows the size of the study area, the number of junctions, the number of nodes, the number of links, and the network density of the three cities. Fig 6(a)–6(c) shows the node distribution and street distribution of the three study areas. Because the links were constructed based on same time-threshold, the differences in network density between the three cities (Table 3 and Fig 6) would influence the connectivity structure of the spatial network. Although the nodes were identified from the streets inside the boundaries of the cities, we included the surrounding areas in calculating the travel times between nodes.

**Table 3:** The summarized network statistics of the three cities.

	Area (*km* ^2^)	Junctions	Settlements	Number of links	Network density	*d*_*min*_ (*km*)	*d*_*max*_ (*km*)
Taipei	270	9244	60	1621	0.92	0.85	15.03
Taichung	2219	18728	60	689	0.39	1.86	21.09
Kaohsiung	2965	16877	60	421	0.24	2.17	18.94

*d*_*min*_: minimum distance between two connected nodes;

*d*_*max*_: maximum distance between two connected nodes.

The parameters, *α* and *β*, in GPR/DDPR algorithms were both set to 1 for initially assessing their performance in the city scales. Fig 6(d)–6(i) shows the results of DDPR (d-f) and GPR (g-i) for the three cities. The patterns of spatial distribution for DDPR and GPR are similar. The distribution of nodes captured the spatial organization of the city’s central-peripheral structure. Spearman’s rank correlations between each of the metrics and village-level population density in each of the three cities are shown in Table 4. Both DDPR and GPR have higher correlations with population density in the three cities than traditional social network metrics. DDPR has a higher correlation than GPR, indicating that distance-decay properties can capture the spatial concentration of human movement. This suggested that distance-decay properties would be sufficient to capture the spatial concentration of human movement.

**Table 4:** The Spearman’s rank correlation (rho) between the network metrics and the population density for the three cities.

	*Degree*	*PR*	*WPR*	*DDPR*	*GPR*
Taipei	0.319*	0.311*	0.305*	0.495***	0.489***
Taichung	0.606***	0.613***	0.600***	0.701***	0.659***
Kaohsiung	0.659***	0.652***	0.662***	0.699***	0.666***

*** significant at 0.001 level,

* significant at 0.05 level.

### The robustness of methodological framework

#### 1. Number of nodes in the K-means clustering procedure

**Fig 7:**
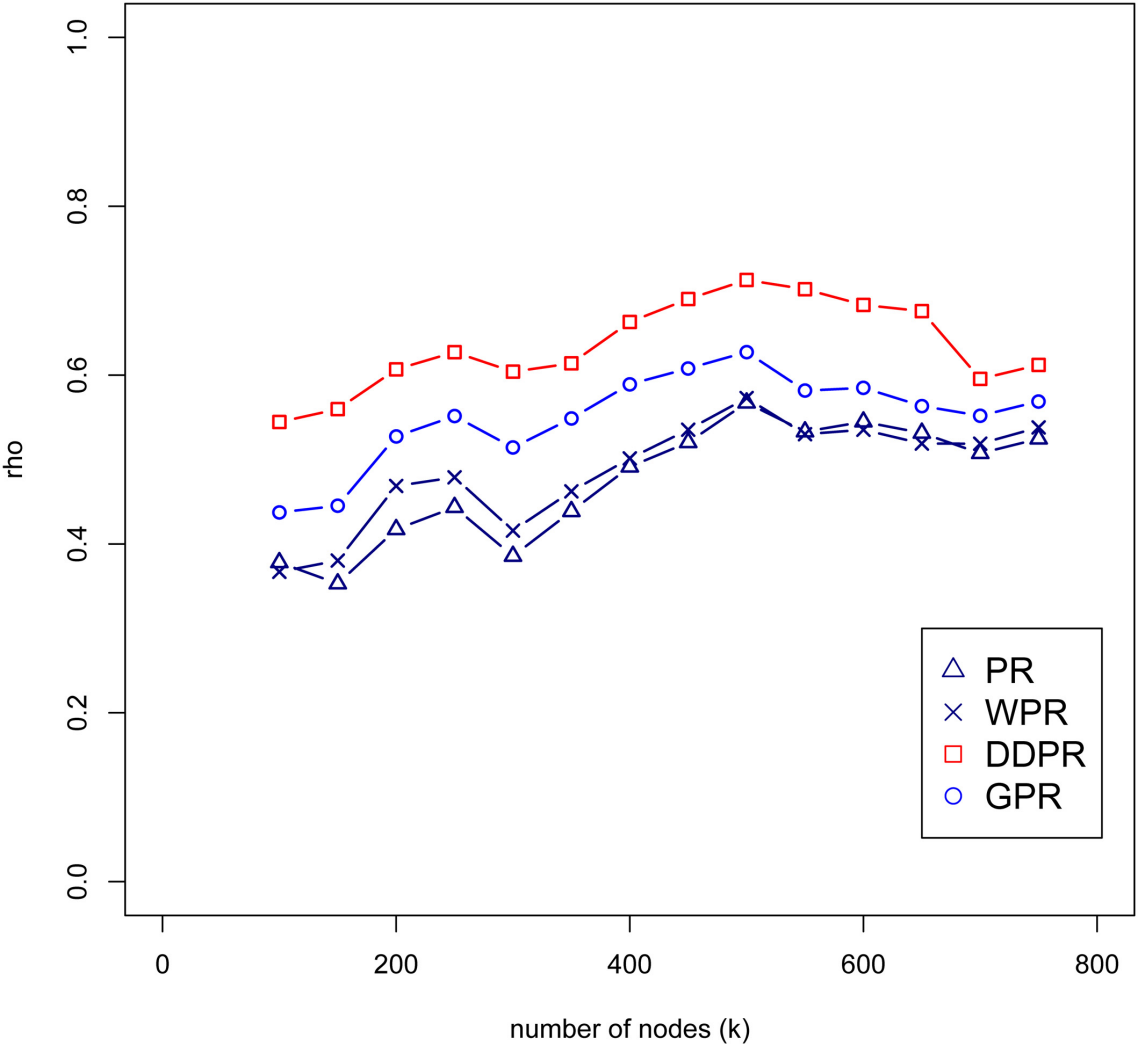
The rank correlation (rho) between different number of nodes (k-value) in range from 100 to 750 and the population density in national scale.

**Fig 8:**
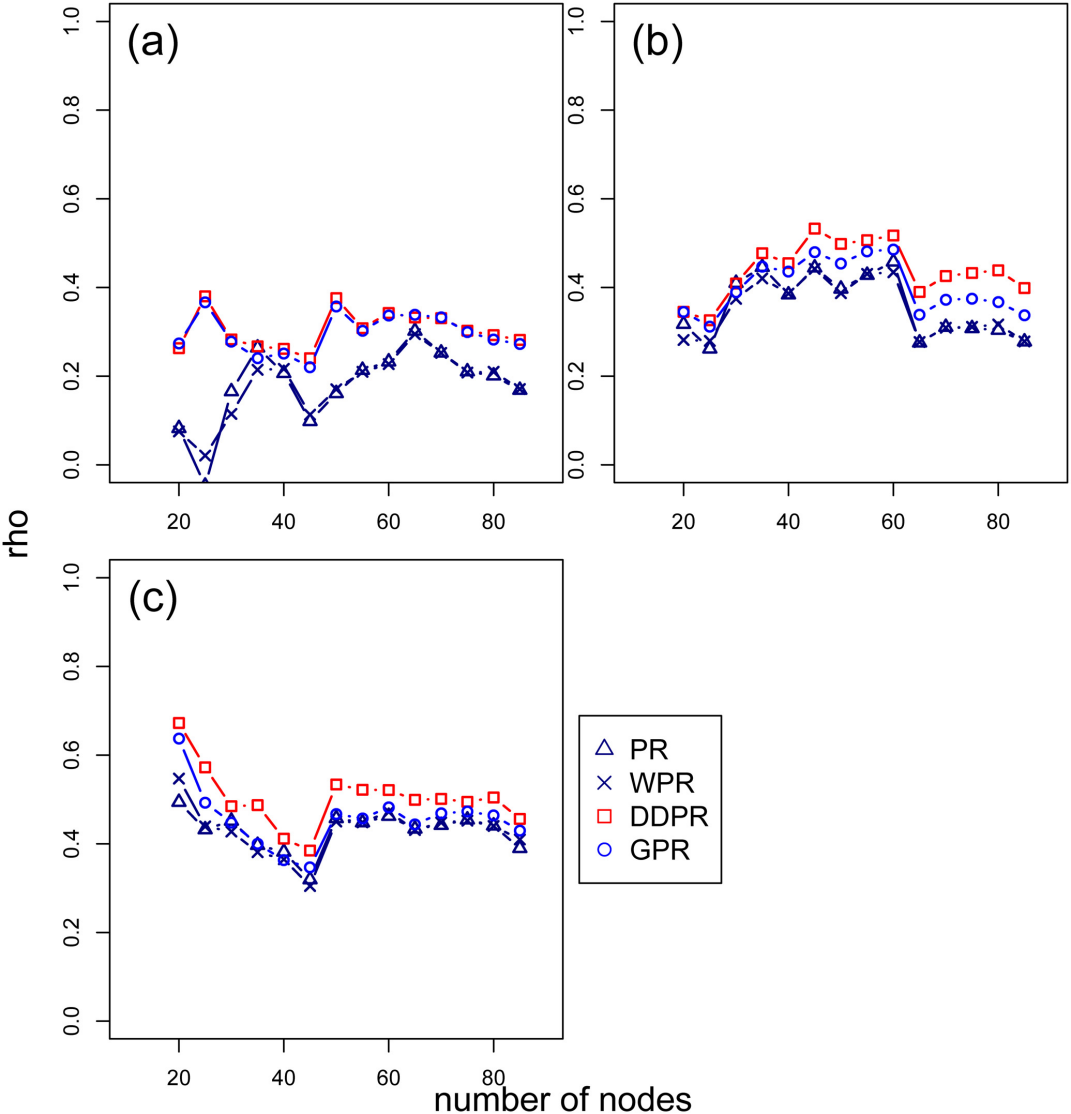
The rank correlation (rho) between different number of nodes (k-value) in range from 20 to 85 and the population density in city scale. (a) Taipei City; (b) Taichung City; (c) Kaohsiung City.

The settlements were used as the nodes in a geospatial network and were identified through the K-means clustering procedure. Based on population, at the national scale the parameter K (number of nodes) was set at 500 (case study 1); at the city scale it was set to 60 (case study 2). To assess the impacts of the chosen k-value on the model performance, we performed a sensitivity analysis setting the k-value in ranges from 100 to 750 for the national scale network and calculated the rank correlation of these results with population density. The results indicate that correlation of increasing k-value between the four PR algorithms (Fig 7) were consistent. Among the 4 PR algorithms, the correlation results between DDPR and population density were higher than the other PR algorithms, following by GPR. In city-scale network, the k-value was set ranging from 20 to 85. The results showed similar findings as in the national scale network (Fig 8). In Taipei, the DDPR and GPR algorithms had similar rank correlation; whereas the rank correlation between PR and WPR was also similar. This was because the number of links of each node is similar, which indicated that the attractiveness of each nodes were similar. In other words, attractiveness is not a significant factor in Taipei network. In Taichung and Kaohsiung networks, the rank correlations were similar to the national scale network. The DDPR and GPR algorithms had the highest correlation with the population density. In summary, the parameter k in the K-means clustering procedure is insensitive to the rank of DDPR and GPR scores in our study.

#### 2. Distance factor in the power-law function

**Fig 9:**
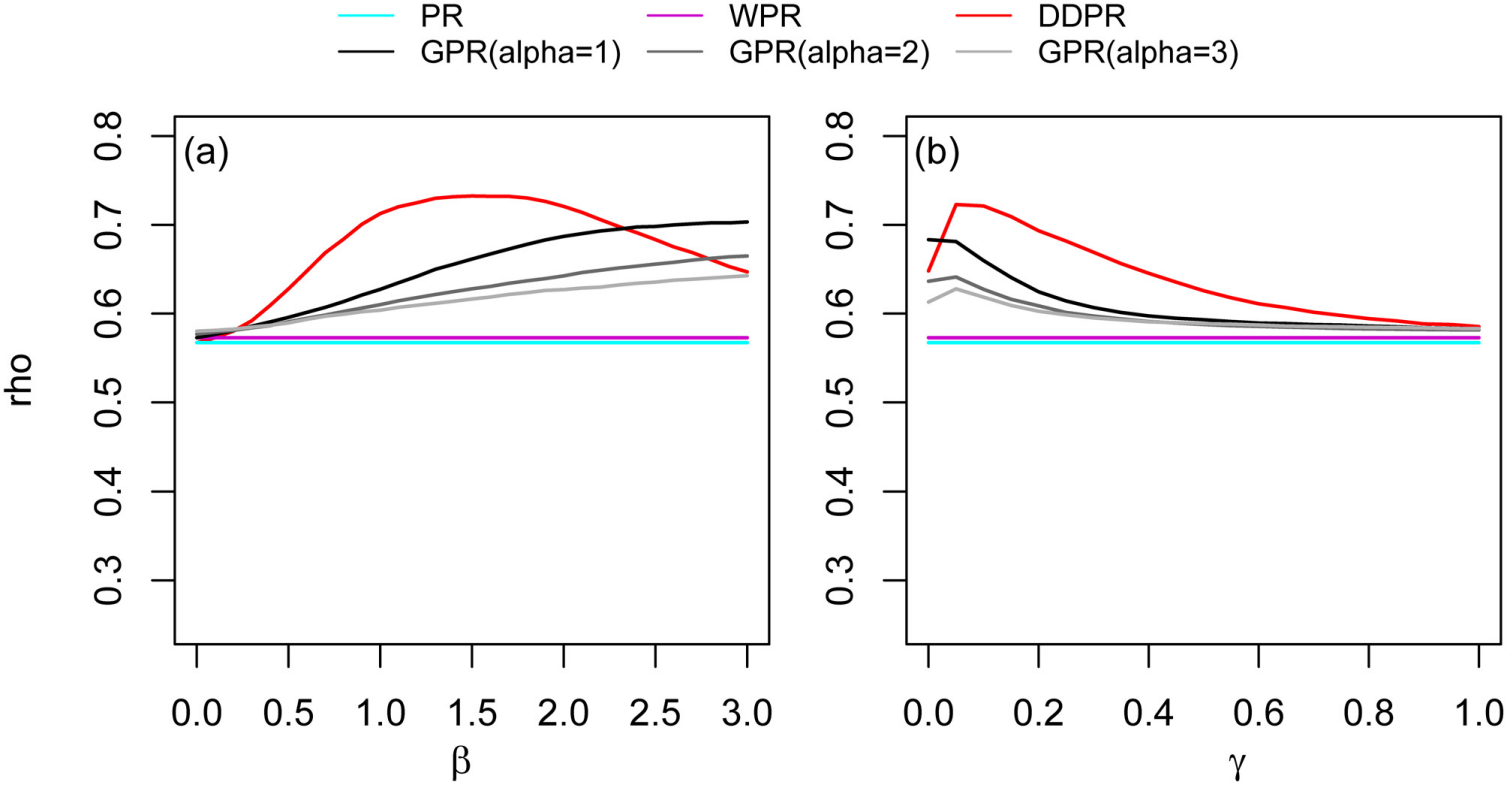
The rank correlation (rho) between the population density and the different (a) *β* and (b) *γ* values in national scale.

**Fig 10:**
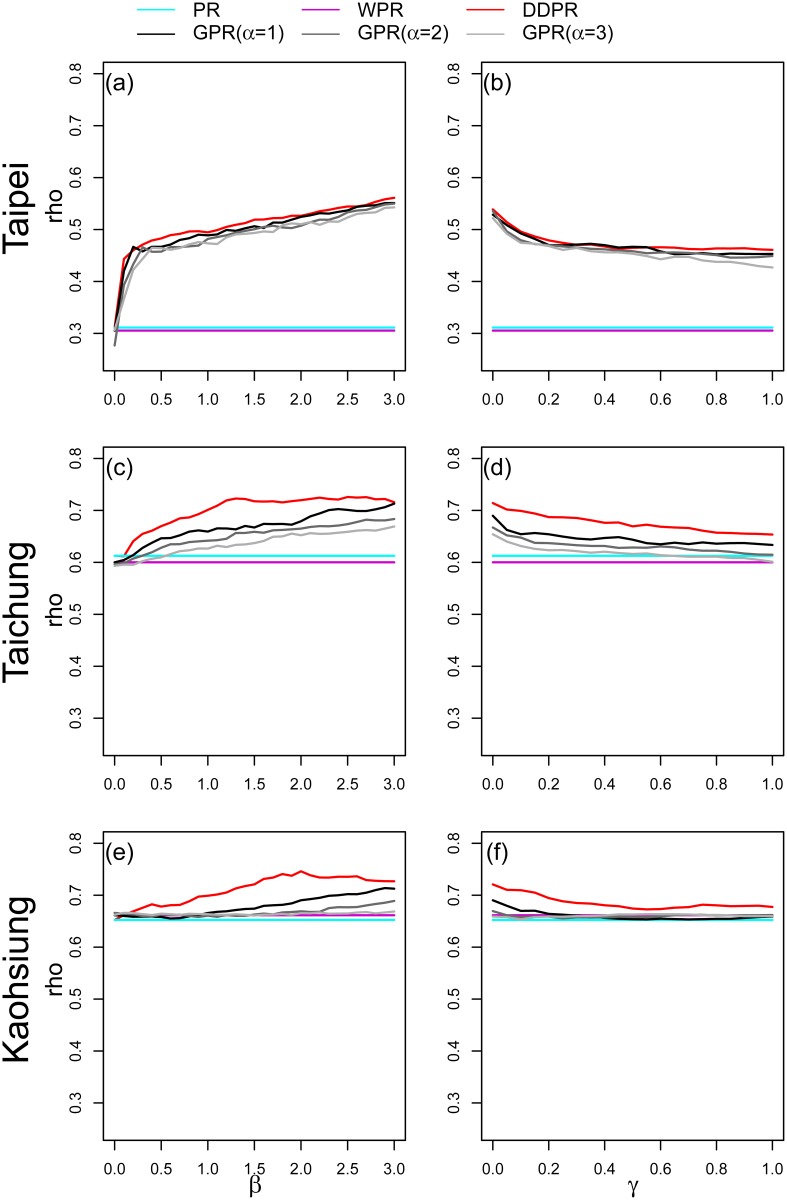
The rank correlation (rho) between the population density and the different values of *β* (left) and *γ* (right) in the three cities.

Fig 9a and the left panel of Fig 10(a), 10(c) and 10(e) show the rank correlation of using different the values of distance factor in the power-law function for the national-scale and city-scale networks respectively. These figures show that the correlation with population density reach the highest Spearman’s rho while *β* is set to 1.5 in national scale, *β* is set to 2.4(for Taichung) and *β* is set to 2(for Kaohsiung) in city scale. In both national and city scale, DDPR (which is a special case of GPR with *α* = 0) is better correlated with population density than GPR with *α* > 0 in most *β* settings, except only while *β* ≥ 2.4 in national scale. The associations in Figs 9 and 10 were statistically significant at 0.05.

#### 3. Distance-control factor in the exponential function

Fig 9b and the right panel of Fig 10(b), 10(d) and 10(f) show the rank correlation between population density and the different value of *γ* in exponential function for the national-scale and city-scale networks respectively. The results in all cases show better correlation results with lower *d*_*γ*_ settings, especially in city scale. In all of the three cities, the correlation lines of DDPR start dropping from the beginning, which is *γ* = 0 (*d*_*γ*_ = *d*_*min*_). In national scale, the correlation line of DDPR reaches the highest point on *γ* = 0.05 (*d*_*γ*_ = 6.97*km*). After that, the correlation line start decreasing until *γ* = 1. This pattern is similar to the correlation lines in the three cities. The correlation lines have similar trend between different settings of *α*, suggesting that *α* is not a sensitive parameter in GPR with exponential decay function. In the comparison between *α*, the correlation result with *α* = 0 (DDPR) holds the best correlation, and the correlation line is lower while *α* is set to be higher.

#### 4. Distance-decay functions: power-law vs. exponential functions

We compared power-law and exponential functions to assess the influence of distance-decay functions on the model performance. Similar to the previous tests, the experiment on distance-decay functions was also performed in both national and city-scale networks. We compared the distance decay functions between their optimal parameter settings in Tables 5 and 6. In overall cases, the DDPR score is better correlated with the population density than GPR one. In comparison between power-law decay and exponential decay functions, the modified PR scores (DDPR and GPR) with power-law decay functions showed better correlation with the spatial concentration of human movement in their optimal parameter settings.

**Table 5:** The optimal parameter settings and correlation results of DDPR with power-law and exponential decay functions.

case	*β*′	*γ*′	$d_{\gamma }^{\prime }(km)$	DDPR	
				power-law	exponential
Taiwan	1.5	0.05	6.97	0.732***	0.723***
Taipei	3.0	0.00	0.85	0.561***	0.539***
Taichung	2.4	0.00	1.86	0.726***	0.714***
Kaohsiung	2.0	0.00	2.17	0.746***	0.721***

*** significant at 0.001 level.

**Table 6:** The optimal parameter settings and correlation results of GPR with power-law and exponential decay functions.

case	*β*″	*γ*″	$d_{\gamma }^{\prime \prime }(km)$	GPR(*α*″)	
				power-law	exponential
Taiwan	3.0	0.00	6.97	0.703***(1)	0.683***(1)
Taipei	3.0	0.00	0.85	0.551***(1)	0.536***(2)
Taichung	3.0	0.00	1.86	0.713***(1)	0.690***(1)
Kaohsiung	2.9	0.00	2.17	0.714***(1)	0.690***(1)

*** significant at 0.001 level.

## Discussion

With only relied on simplified transportation network (streets and railways) as a geometric graph to represent urban connections, our study captured the spatial distribution of population and mobility flows. Our results showed that the topological structure of connectivity and accessibility between places could reflect the locations where people tend to agglomerate. It implies that spatial constraints are one of the important factors for understanding routine population movement [2], and extending the PR algorithms with the distance-decay properties and attraction properties is necessary for assessing the connectivity and critical nodes of a geospatial network. In case of the concentration of human movement, the geographical proximity is an important factors [17] [18]. On the other hand, PR and WPR were designed to identify the network important nodes from a pure topological network, which were lack of the consideration of the spatial proximity effect and thus not suitable for suitable for exploring the population movement network.

This study proposes two algorithms—the Distance-Decay PageRank (DDPR) and the Geographical PageRank (GPR)—to capture the concentration of human movement in a geospatial network, abstracting from a transportation network. Our results show that the concentration of human movement are better correlated with DDPR and GPR scores in comparison with traditional network metrics. This finding suggests that the DDPR and GPR algorithms can effectively capture the spatial locations where people reside. In addition to network connectivity, geographic considerations, including distance-decay properties and location attraction, also help to determine the spatial concentration of human movement in a geospatial network [7] [10]. Previous studies have shown that the number of interactions between people, including message delivery in an online community from ones’ geographic location to a friend’s geographic location [39] and mobile phone calls between people at different locations [40] [41], tend to follow distance-decay rules. In this study, we have found that network metrics that incorporate the distance-decay effect can capture spatial demographic patterns between spaces better than metrics that do not account for distance. Furthermore, in comparison with national-scale intercity and city-scale connections of geospatial networks, both DDPR and GPR scores exhibit high correlations with spatial concentration of human movement in different spatial scales. This suggests that geographic considerations may have cross-scale influence on spatial concentration of human movement. Geographic considerations have been used as a necessary ingredient in exploring relationships within spaces of various scales, for example, transportation costs, distance, tariffs and economic status in international trade [42] [43], numbers of immigrants and emigrants, the economic and demographic status of destinations in international migration [44] [45], sizes of commuting flows between cities [46] [47], and traveling distances and riderships in movement within a city [48] [49] [50].

The difference between DDPR and GPR is that GPR includes both location attractiveness and the distance-decay effect, whereas DDPR only consider the distance-decay effect. Nodal attractiveness represents the potential of a location, i.e., how effectively a node drives people or resources toward it. WPR is another modified PR algorithm, proposed by Xing and Ghorbani [22]. This algorithm only considers nodal attractiveness, so that additional PR scores are sent to nodes that are more attractive. Our results show that with respect to national-scale connections, WPR is better correlated with human movement concentration than PR is (Table 2). This result is similar to the findings of Jiang [7], who showed that WPR is a better human movement predictor than PR scores. However, when nodal attractiveness and the distance-decay effect are incorporated into GPR, GPR exhibits better statistical performance than WPR (Table 2). In DDPR, only the distance-decay property affects the distribution of PR score transmissions between nodes. Although WPR and GPR are better correlated with spatial concentration of human movement than traditional PR and other network metrics, our results show that DDPR exhibits even higher correlations than the WPR and GPR (Table 2). This finding suggests that the distance-decay effect is a more important factor than nodal attractiveness in determining rankings of concentration of movement. Our findings demonstrate that the effect of distance remains significant on the commuting network at various scales [39] [50] [51] [52]. The results in Figs 9 and 10 show that DDPR is better correlated with the concentration of human movement than GPR. These results indicate that the findings in initial settings (*α* = 1 and *β* = 1) are consistent in both scales. Therefore, we suggest that DDPR could be sufficient and desirable for capturing the concentration of human movement.

The correlation results for the three cities consistently show that DDPR has the highest correlation with spatial concentration of human movement, whereas GPR has the second highest correlation, suggesting that the results at the city scale resemble the results at the national scale (Table 4 and Fig 6). Among the three cities, the differences of rank correlation were determined by their attractiveness and distance-decay relationship. The attractiveness was defined by the in-degree of a node. As a result of Taipei’s well-developed mass rapid transport system, the population mobility breaks the space-time constraints and it causes the decrease in distance-decay effect. Therefore, the population density of Taipei is difficult to be captured by only using topological measures. On the other hand, Taichung and Kaohsiung cover the areas with mix and heterogeneous development levels. Network structure could reflect the population distribution in these cities (Table 3). Therefore, topological measures in Taichung and Kaohsiung showed better performance in terms of rank correlation than Taipei city.

The weighting scheme in PR algorithms was using the weight of target and comparing it to the other destination originated from the source, to decide the probability of a random surfer moving from the source to the target (PR could be understand as a special case of WPR that all node has the same weight). This scheme is also similar to the concept of radiation model, which is an alternative of the gravity model to understand human movement. Radiation model is a parameter free spatial model, which suggested that the flow from a source to a target area depends only on the population of the two areas, and the population of the other areas whose distance from the source is less than the distance from the source to the target area [53]. The radiation model compares the target population to the population of other potential destination originated from the source and the interaction between the population of the potential destinations, to measure the strength of the links from the source to the target, which were than used to calculate the probability of a particle (moving agent) to be absorbed by the target from the source [54]. By using similar weighting scheme, WPR used the attractiveness of the target and compared it to the other potential destination. This means if there exist another destination whose attractiveness is same as the target, they would share the same probability, even if the second destination is farther away than the target from the source. Thus, the integration with the distance-decay function is necessary to capture the proximity differences between the destinations. Hence, GPR, which considered both attractiveness and distance-decay effects, could be an alternative to radiation model in a geospatial network analysis. Moreover, regarding the modeling of spatial interactions among areas, moving agents could followed certain rules to be absorbed by any destination in radiation model; random surfers could only move within the network through the links in PR-family algorithms, and the probability of moving on each links depends only on the outgoing links of the source. The outgoing links are the only options for surfer moving from current location. The PR-family algorithms could reflect more realistic movement trajectories.

On the other hand, global-wide urban road network data is openly and readily available from internet database, such as OpenStreetMap project. Our algorithm could be more applicable than past extend models when we incorporated the link-node structures of road network and railways as a geometric graph to represent urban connections. In our study, geographic distance is formulated as spatial constraints in terms of distance-decay weighting scheme for measuring population mobility in national and city scales. Our results showed that the extended PR algorithm with the distance-decay properties and attraction properties captured the spatial patterns of population distribution. Long travel distance could still act as impedance on population movement causing the decrease of interactions between areas even in the era of information and communication technology.

This study has several limitations. First, types of nodes and links have been neglected. Because nodes represent different locations, some nodes may be residential areas with dense populations, some may be business areas with high volumes of traffic, and some may be industrial areas with massive man-made facilities. Therefore, different types of nodes could function differently in the geospatial network. Second, we simplified nodal attraction, using the numbers of connections to each node. Methods of capturing nodes’ attractiveness could be varied based on the type of location each node represents, for example, major land-use type, nearby facilities and quantity of employment opportunities [5] [10] [53]. Additional approaches to nodal attraction may improve the correlation results, but they would also increase the complexity of the analysis. Third, in addition to node characteristics and nodal attraction, we also simplified connection types and capacities with bidirectional and unweighted links. Although the algorithm was designed to explore the reachable relationships between spaces, the mode of movement and the number of paths could affect connectivity and the strength of links. Thus, the algorithm does not differentiate effects related to types and capacities of connections. These issues warrant further investigation.

## Conclusion

Geographic proximity and location attractiveness are important spatial factors in measuring the importance of locations. Geographically modified PageRank algorithms—Distance-Decay PageRank (DDPR) and Geographical PageRank (GPR), which incorporate geographic considerations into the PageRank algorithm—have been proposed as methods to identify the spatial concentration of human movement within a geospatial network. At both the national scale and city scale, these proposed algorithms are more effective at capturing spatial patterns of human residence than other commonly-used network metrics. In comparing location attractiveness with distance-decay effects, we conclude that the spatial concentration of human movement is dominantly determined by distance-decay effects, which implies that geographic proximity remains a key influence on human mobility.
